# Establishing scientific confidence in a two-chamber co-culture system to evaluate androgenic response in the presence of hepatic metabolism

**DOI:** 10.1016/j.toxlet.2025.08.014

**Published:** 2025-09-01

**Authors:** Tessa C.A. van Tongeren, Susan J. Hall, Samantha J. Madnick, Blanche C. Ip, Paul L. Carmichael, Hequn Li, Wei Chen, Lori A. Breitweiser, Heather E. Pence, David M. Ames, Andrew J. Bowling, Kamin J. Johnson, Richard Cubberley, Bruce Sherf, Jeffrey R. Morgan, Kim Boekelheide

**Affiliations:** a Division of Toxicology, Wageningen University and Research, Wageningen 6700 EA, the Netherlands; b Department of Pathology and Laboratory Medicine, Brown University, Providence, RI, USA; c Center for Alternatives to Animals in Testing, Brown University, Providence, RI, USA; d Unilever Safety and Environmental Assurance Centre, Sharnbrook, Bedfordshire MK44 1LQ, UK; e Corteva, Inc., Indianapolis, IN, USA; f INDIGO Biosciences, Inc., State Collage, PA, USA

**Keywords:** Toxicity testing, 3D HepaRG microtissues, AR-CALUX, AR-INDIGO, *in vitro* testing

## Abstract

For the *in vitro* determination of toxicity on target organs in the presence of physiologically relevant human metabolism, we recently developed a two-chamber liver-target organ co-culture system in a medium-throughput 96-well format. Our proof-of-concept study using human HepaRG microtissues cultured in three-dimension (3D) and AR-CALUX reporter cells demonstrated the significantly reduced testosterone (T)-mediated androgen receptor (AR) responses in the presence of human liver metabolism. The present study further increased the scientific confidence in this two-chamber co-culture system as a flexible and robust tool to capture androgen-mediated responses by incorporating alternate AR reporter cell systems as the target and examining additional androgenic compounds. The system generated concordant metabolism-dependent changes in T- and 5α-dihydrotestosterone (DHT)-mediated AR responses using two different AR reporter cell systems (AR-CALUX, AR-INDIGO). The AR reporters had different sensitivity ranges and required media optimization. We demonstrated that this two-chamber co-culture system with integrated hepatic biotransformation can be used to evaluate endocrine activity with potential metabolism modulation of parent compounds.

## 1. Introduction

In next generation risk assessment (NGRA), new approach methodologies (NAMs) are developed to replace, reduce, and refine (3Rs) animal use for experimentation (Russell & Burch, 1959) to assure chemical safety in humans (US EPA, 2018; Punt et al., 2020; Parish et al., 2020). NAMs include *in vitro* bioactivity assays to quantify toxicodynamic responses of chemicals in cells. *In vitro*-derived point of departure (PoD) can be defined to set safe exposure levels of chemicals (Baltazar et al., 2020; P. Carmichael et al., 2009; P. L. Carmichael et al., 2022; Middleton et al., 2022; Chang et al., 2022; Wambaugh et al., 2025). However, *in vitro* cell lines seldom capture the kinetics of a complex *in vivo* environment where hepatic metabolism can transform compounds to either more or less active metabolites. Consequently, the role of potential (in) active metabolites is often missed in the quantified *in vitro* response (Coecke et al., 2006; Gu and Manautou, 2012; OECD DRP 97, 2008; Ooka et al., 2020), compromising the predictive power of the assay.

For example, our understanding of the testosterone (T)-mediated androgen receptor (AR) response measured in an *in vitro* reporter gene assay is incomplete without considering that T is hepatically activated to dihydrotestosterone (DHT) and both T and DHT are inactivated by metabolism. Thus, quantifying the androgenic response of T or DHT with *in vitro* AR reporter gene assays does not provide a physiological reflection of the response at the *in vivo* target site since, in the absence of metabolism, activating and inactivating reactions do not occur. The AR response is essential in the development and maintenance of the male reproductive system (Marcoccia et al., 2017; Schiffer et al., 2018), thus *in vitro* characterization is important when using NAMs to study potential androgenic effects. To include both parent chemicals and their metabolites in physiologically relevant proportions and with appropriate temporal dynamics in an *in vitro* assay is central to improving predictive toxicology and safety assessments.

**Fig. 1.**
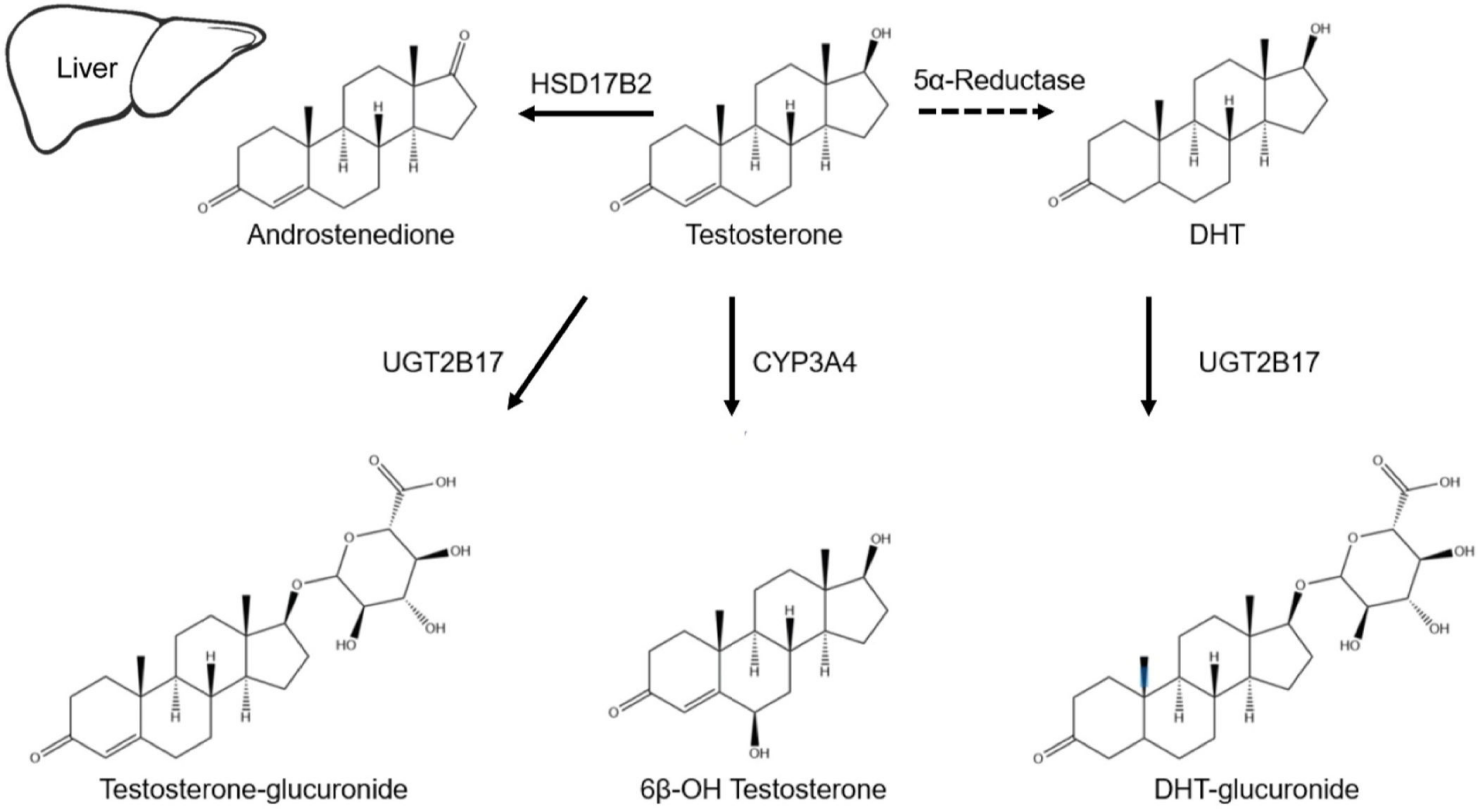
Simplified metabolic pathway of testosterone and dihydrotestosterone (DHT) biotransformation in the liver. The predominant activating (solid lines) and inactivating (dashed line) conversions are depicted: SRD5A1 = 5α-reductase type 1, HSD17B2 = 17β-hydroxysteroid dehydrogenase 2, SRD5A1 = 5α-reductase type 1, GT2B17 = UDP glucuronosyltransferase Family 2 Member B17, CYP3A4 = Cytochrome P450 Family 3 Member A4.

The metabolism of T is well described in the literature and a schematic view of the main pathways is summarised in Fig. 1. DHT is a more AR bioactive metabolite of T, converted by 5α-reductase (SRD5A1). Inactivation pathways in the liver include the oxidation of T to 6β-hydroxy testosterone (6βOHT) predominantly by the cytochrome P450 Family 3 Member A4 (CYP3A4) (Hashimoto et al., 2016; Usmani and Tang, 2004) and oxidation of T to androstenedione (AD) by 17β-hydroxysteroid dehydrogenase 2 (HSD17B2) (Hilborn et al., 2017; Suzuki et al., 2000; Wilson and LeBlanc, 2000). DHT and T are conjugated mainly by UDP-glucuronosyltransferase Family 2 Member B17 (UGT2B17) yielding T- and DHT-glucuronide (TG and DHTG) excreted in urine (Bhatt et al., 2018; Li et al., 2019; Schiffer et al., 2018; Usmani and Tang, 2004; Zhang et al., 2018).

Hepatic biotransformation of compounds *in vitro* can be measured by incubation in suspensions of primary human hepatocytes (PHHs), which are considered the gold standard *in vitro* liver model (Jackson et al., 2016; Stanley and Wolf, 2022). However, PHHs have significant limitations for use in *in vitro* studies as reproducible and robust tool, including availability of human liver tissue, large inter-donor variations in enzyme levels, and a limited *in vitro* half-life for expression of their differentiated functionality (Ramaiahgari et al., 2017). A more stable *in vitro* alternative to measure biotransformation is provided by the immortalized human hepatocellular carcinoma-derived HepaRG cell line, which can be differentiated *in vitro* to hepatocyte- and cholangiocyte-like cells (Aninat et al., 2006; Gripon et al., 2002). Three dimensional (3D) HepaRG microtissues have prolonged and more physiologically relevant liver functionality as compared to 2D HepaRGs, including physiological Phase I and Phase II enzyme levels and activities, a biliary excretion system, and hepatic zonation characteristics under different medium conditions (Gunness et al., 2013; Jackson et al., 2016; Leite et al., 2012; Ramaiahgari et al., 2017; Ip et al., 2024). 3D HepaRG microtissue formation can be achieved by culturing the cells in a non-adhesive agarose mold system which creates a stable microenvironment (Ip et al., 2018, 2022) in which chemicals can freely diffuse (Ip et al., 2024).

Ip et al. (2024) developed a two-chamber liver-target organ co-culture model in a medium-throughput 96-well format for the determination of toxicity on target tissues in the presence of physiologically relevant human liver metabolism, utilizing 3D HepaRG microtissues as the source of liver biotransformation. As a proof-of-concept, the androgen receptor (AR)-CALUX (Chemically Activated LUciferase gene eXpression) reporter assay (Sonneveld et al., 2005; van der Burg et al., 2010) was used as the target cells to determine androgenic responses of T in the presence of physiologically relevant human liver metabolism. This two-chamber co-culture system, based on agarose, allows 3D HepaRG microtissues to be formed and cultured in a space separate from the AR reporter gene target tissue, but allows the free diffusion of metabolites between chambers. Prostate stromal and epithelial cells and estrogen receptor reporter cells were also confirmed compatible in the system. This proof-of-concept work demonstrated the efficacy of the co-culture system to evaluate T-mediated AR responses in the presence of human liver metabolism at physiologically relevant testosterone concentrations (10–1000 nM). Half of the T was metabolized within 24 h with a 17 % conversion to androstenedione (AD). This result is in concordance with the detoxification of T by generating the less androgenic AD metabolite (Sonneveld et al., 2005).

The aim of the present study was to evaluate the flexibility and the robustness of the two-chamber co-culture system by including another androgenic reporter cell line and to compare the corresponding responses to those obtained with the AR-CALUX cells. To this purpose we selected the human AR-INDIGO reporter cells, an androgen responsive cells that also uses a luciferase reporter gene (INDIGO Biosciences, Inc., State Collage, PA, USA). AR-CALUX cells are a human osteosarcoma cell line U2OS that was stably transfected with the luciferase gene, whereas the AR-INDIGO cells are based on a different cell type (CV-1, isolated from the kidney of a male adult African green monkey) and are transiently transfected to achieve high level expression of the luciferase gene. Lastly, the INDIGO platform has a wide range of applications with reporter cells that have been engineered to report cell viability, cytokine signalling, growth factor signalling, nuclear receptor activity GPCR signalling as well as the activity of transcription factors, all of which could potentially be modified by liver metabolites of an environmental chemical or drug candidate. Both T and DHT were used as model compounds to cover broader androgenic biotransformation reactions. Extensive evaluation of co-culture assay conditions was conducted to investigate how media composition and 3D HepaRG maturation time impacted biotransformation of this co-culture system, using T and DHT as the test compounds.

## 2. Material and methods

### 2.1. Materials

Dulbecco’s modified Eagle’s Medium/Ham’s nutrient mixture F12 (DMEM/F12, 10565–018), GlutaMax (35050061), penicillin/streptomycin (15140–122), MEM (100 ×) non-essential amino acids (NEAAs, 11140035), Geneticin (G418, 10131035), phosphate buffered saline (PBS, 14190), trypsin EDTA (trypsin (0.025 %)/EDTA (0.01 %), 15400–054), UltraPure agarose (BP160–500), phenol red free Williams’ E medium (A12176–01), dimethyl sulfoxide (DMSO, BPBP321–100), trypan blue (T10282), Triton (X100), and LIVE/DEAD™ Viability/Cytotoxicity Kit, for mammalian cells (L3224) were purchased from ThermoFisher (Fisher Scientific, Waltham, MA, USA). Testosterone (CAS no. 58–22–0, T1500), DHT (CAS no. 521–18–6, A8380), human insulin (I9278), and hydrocortisone (HC, H088) were purchased from Sigma (MilliporeSigma, Burlington, MA, USA). Charcoal dextran stripped fetal bovine serum (CDS-FBS, 100–119) was purchased from GeminiBio (West Sacramento, CA, USA). MHTAP HepaRG supplement was purchased from Lonza (Basel, Switzerland). Lysis mix (Cat no: 26) and Illuminate mix (Cat no: 35) were purchased from BioDetection Systems (BDS, Amsterdam, The Netherlands). Cell recovery medium (CRM) was donated by INDIGO Biosciences Inc. (State College, USA). 96-square well plates (50305829) were purchased from Ibidi (Gräfelfing, Germany). LoBind Protein tubes (0030108442) were purchased from Eppendorf (Hamburg, Germany). The materials used for the mRNA analysis, immunohistochemical analysis, and LC-MS/MS analysis were previously reporteded by Ip et al. (2024).

## 3. Methods

### 3.1. Fabrication of two-chamber co-culture system in 96-well plate platform

**Fig. 2.**
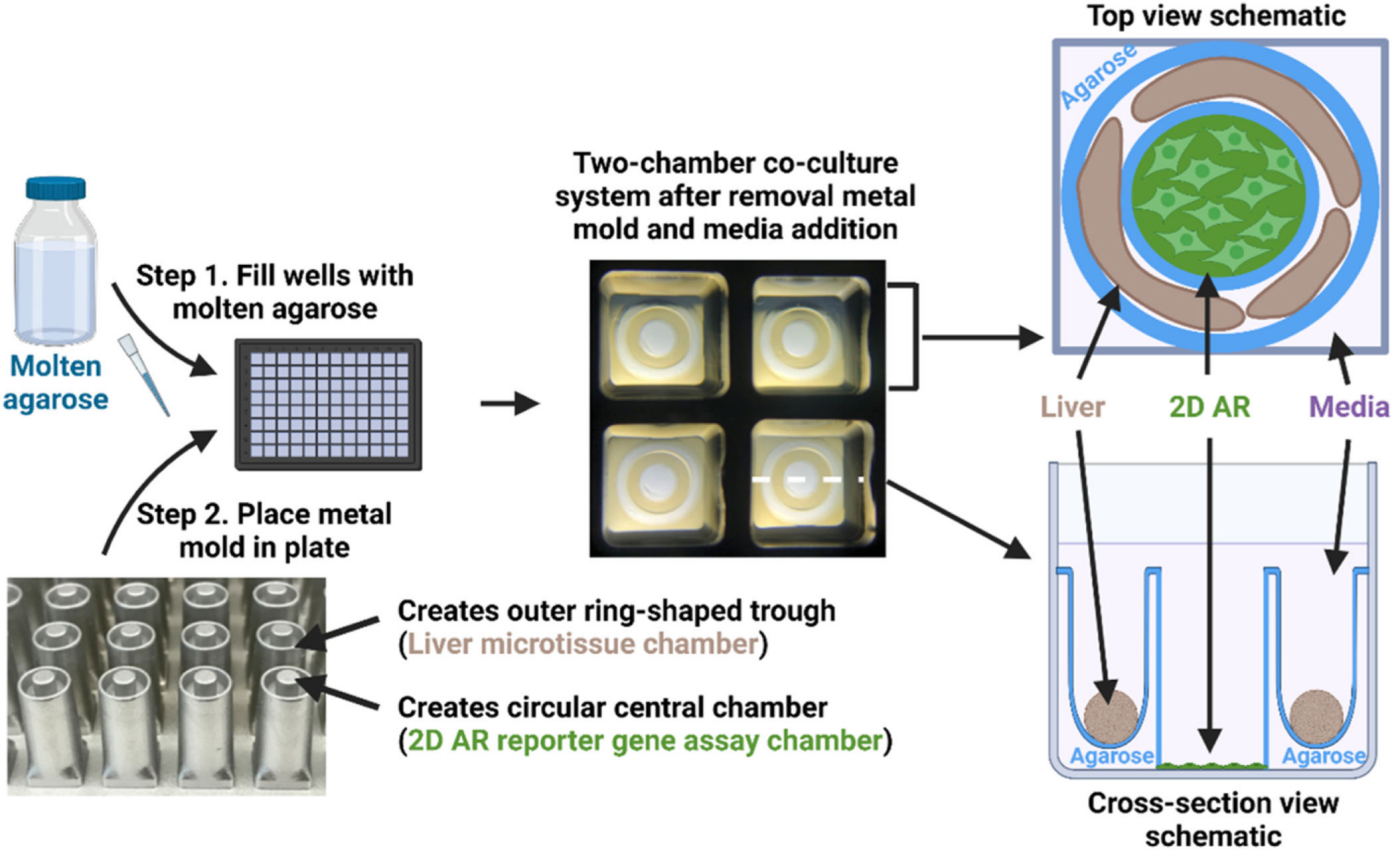
Schematic representation of the development of the two-chamber co-culture system in a 96 square well plate format. In Step 1, molten 2 % agarose hydrogel is added to the wells of the plate and in Step 2 the metal mold is inverted in the plate until the agarose hardens. After removal of the metal mold and addition of medium, the outer ring-shaped trough is seeded with HepaRG cells and the circular central chamber is seeded with AR reporter cells that adhere to the bottom of the plate (adapted from Ip et al., 2024). Medium is added by placing the pipette tip in the corner of each well, filling and topping off both chambers with medium. The right panel displays a schematic top and cross-sectional view of the agarose mold with HepaRG microtissue and AR tissue in one well.

The two-chamber co-culture system using agarose hydrogels was fabricated as described by Ip et al. (2024) in Fig. 2. In short, a stainless-steel mold was designed using computer-assisted design (CAD) (Solidworks, Concord, MA) and consisted of a base platform with a series of pegs each with a circular ring and a central cylinder-shaped peg that touches the bottom of the plate when the mold is inserted. The mold was designed to fit in a 96-square well plate (Ibidi Gräfelfing, Germany). To make the agarose hydrogel-based two-chamber system in the wells, each well was filled with 135 μL sterile molten agarose in sterile PBS (2 % w/v) whereafter the heated stainless-steel mold was placed inverted into the plate. After the agarose solidified, the stainless-steel mold was removed, leaving an agarose hydrogel with an outer ring-shaped trough (HepaRG cells chamber) with a circular central chamber at the bottom of the plate (AR reporter gene assay chamber). Wells were equilibrated with sterile PBS with 1 % penicillin/streptomycin and stored at 4°C until the plates were ready to be used. The plates were prepared for experimentation by equilibrating 3 times every 2 h with 200 μL base medium consisting of serum-free Williams’ E medium supplemented with 1 % GlutaMax and 1 % penicillin/streptomycin, and placed in an incubator (37°C, 5 % CO_2_, 100 % humidity). The wells around the periphery of the plates devoid of the agarose hydrogels were filled with sterile PBS with 1 % penicillin/streptomycin to prevent evaporation in the inner wells, leaving 60 wells available for experimentation.

### 3.2. Differentiation of HepaRG cells

HepaRG cells were expanded and differentiated according to the supplier’s protocol (BioPredic International, HPR101, Rennes, France; proprietary method). Differentiated cells (6.5 million cells per vial) were frozen by placing the cells overnight at −80°C with Mr. Frosty™ Freezing Containers (5100–0001) and then stored in liquid nitrogen.

### 3.3. Formation of 3D HepaRG microtissues in the two-chamber co-culture system

On day 0 (D0), differentiated HepaRG cells were thawed in 4.5 mL base medium supplemented with MHTAP HepaRG (MHTAP medium), centrifuged at 500 x *g* for 3 min (Avanti J-14 Centrifuge, Beckman Coulter), reconstituted in MHTAP medium after which live cells were counted with the Nageotte Counting Chambers (Hawksley, United Kingdom). Serum-free medium was aspirated from the wells with agarose hydrogels and the differentiated HepaRGs were seeded at a density of 50,000 cells in a volume of 25 μL MHTAP medium per well in the outer ring-shaped trough of the agarose two-chamber system. The central chamber was left empty. Note that all pipetting steps were executed slowly to avoid cells drifting to the other compartment. After a 20 min settlement of the cells, 150 μL of MHTAP medium was added to the wells, filling up both the outer ring-shaped trough and central chamber with medium and topping them off. Plates were incubated (37°C, 5 % CO_2_, 100 % humidity) on an orbital rotator (36 rpm, Orbi-Shaker™ CO_2_, Benchmark Scientific, Sayreville, NJ) overnight, wherein the HepaRGs formed 3D HepaRG microtissues.

### 3.4. HepaRG microtissue characterization

All experimental procedures described in the next sections are summarized in Supplementary materials S1. For HepaRG microtissue characterization, on day 1 (D1) after visual examination of the microtissues, 140 μL medium was replaced with 140 μL differentiation medium (DM, consisting of base medium supplemented with 10 % CDS-FBS, 5 μg/mL human insulin, and 0.5 % DMSO) maintaining differentiation. On day 2 (D2), 100 μL DM was refreshed. On day 3 (D3), brightfield images were obtained for further visual evaluation with the Perkin Elmer Opera Phenix taking images every 30 μm with a 5x air objective to generate stitched maximum projection images using the Perkin Elmer Harmony software (Harmony 4.9; Perkin Elmer). This allowed assessment of a shorter maturation time of 3 days as opposed to 10 days (Ip et al., 2024) and different media conditions on the formation of 3D HepaRG microtissues.

### 3.5. Gene expression analysis in 3D HepaRG microtissues

Gene expression analysis was completed with HepaRG microtissues at 5 and 24 h following the medium change to DM (at D1 +5 h and D2, respectively). The plate containing the HepaRG microtissues was placed on ice and the microtissues were collected for RNA extraction. The medium was aspirated and the wells were washed three times with ice-cold PBS. The HepaRG microtissues from the outer ring were transferred into 1.5 mL Eppendorf tubes with a wide bore pipette tip. Tissues were then centrifuged at 600 x *g* at 4°C for 10 min (Avanti J-14 Centrifuge, Beckman Coulter). The supernatant was removed and tubes were frozen in liquid nitrogen. Tissues were stored at −80°C until analysis for the gene expression of CYP1A1, CYP1A2, CYP2B6, CYP2C9, CYP3A4, and UGT2B17. From the frozen HepaRG microtissues, RNA was extracted as described (by Ip et al. (2024). For the analysis, probes for genes of interest (GOI) were included: CYP1A1 (Hs01054796_g1), CYP1A2 (Hs00167927_m1), CYP2B6 (Hs04183483_g1), CYP2C9 (Hs04260376_m1), CYP3A4 (Hs00604506_m1), and UGT2B17 (Hs07293020_g1). Hs01060665_g1 was used for normalization of gene expression. qRT-PCR analysis was conducted as described by Ip et al. (2024).

### 3.6. Immunohistochemical analysis of CYP3A4 in 3D HepaRG microtissues

HepaRG microtissues were immunostained for CYP3A4 (Abcam, ab124921) as described by Ip et al. (2024) to evaluate CYP3A4 protein expression of HepaRG microtissues under different maturation time, media and drug treatments. The following groups were investigated: 1) D1 HepaRG microtissue, 2) D1 HepaRG microtissue switched to DM for 5 h than exposed to 10 nM T, 3 nM DHT, or vehicle control in DM for 24 h, and 3) D1 HepaRG microtissue switched to DM for 24 h than exposed to 10 nM T, 3 nM DHT, or vehicle control in DM for 24 h. All treatments had a final DMSO concentration of 0.29 %.

### 3.7. Cell culture AR-CALUX

Cells from the stably transfected human osteosarcoma (U2OS) cell line expressing the human AR (BioDetection Systems (BDS), Amsterdam, the Netherlands) were maintained in DMEM/F-12 supplemented with 10 % FCS, 1 % NEAAs, 10 units/mL penicillin, 10 μg/mL streptomycin, and 0.2 mg/mL G418 in an incubator (37°C, 5 % CO_2_, 100 % humidity). The cells were routinely subcultured when reaching 85–95 % confluency (*i.e.* every 3–4 days) using trypsin-EDTA (Sonneveld et al., 2005; van der Burg et al., 2010).

### 3.8. AR-INDIGO cells

Proprietary CryoMite™ preserved cells from the human AR transfected African green monkey kidney-isolated CV-1 cell line (NR3C4, INDIGO Biosciences Inc., State College, United States) (INDIGO cells) were stored in the −80°C at a density of 1.5 million cells/mL per vial in a volume of 0.6 mL.

### 3.9. LIVE/DEAD staining of U2OS AR-CALUX and CV-1 AR-INDIGO cells in the central chamber of the two-chamber co-culture system

We used LIVE/DEAD staining (Invitrogen Corp., Carlsbad, United States) to evaluate the viability of the U2OS AR-CALUX and CV-1 AR-INDIGO cells in the central chamber with or without HepaRG microtissues after exposing to T, DHT, vehicle control, or 0.1 % triton in ultrapure water as the positive dead control. 140 μL medium was first carefully removed from each well with or without D1 differentiated HepaRG microtissues, then medium in the central chambers were further aspirated. 8000 AR-CALUX cells were seeded into each of the central chambers of the agarose two-chamber system in 15 μL of DM. To seed the human AR-INDIGO cells, frozen vials of cells were rapidly thawed by adding 6.4 mL pre-heated (37°C) CRM to the vial and placed in a water bath (37°C). After 5–10 min, the AR-INDIGO cells were centrifuged at 500 x *g* for 3 min (Avanti J-14 Centrifuge, Beckman Coulter). The supernatant was removed and cells were resuspended in 1.5 mL CRM. 15 μL of this AR-INDIGO cell suspension was slowly added to the central chamber, achieving approximately 9000 cells per well. The cell amounts slightly differs than that of the AR-CALUX cell due to the number of cells in the frozen vials supplied by INDIGO. After 5–10 min, 140 μL of DM was slowly added to each well containing either AR-CALUX or AR-INDIGO cells to cover both compartments without disturbing the newly seeded cells. The plates were then incubated at 37°C, with 5 % CO_2_ and 100 % humidity for 24 h then exposed either to T or DHT at different concentrations or vehicle control for 24 h, or 0.10 % triton in ultrapure water for 24 h (AR-CALUX cells) or 10 min (AR-INDIGO cells). The different 0.10 % triton exposure time was required to optimize staining for each of the reporter cell lines. For LIVE/DEAD staining, medium was aspirated and the agarose hydrogels were removed using a pipette tip. Cells were washed 2 times with PBS then stained with 100 μL of LIVE/DEAD reagent (AR-CALUX cells: 1 μM ethidium homodimer (EthD-1) and 0.5 μM calcein acetoxymethylester (AM) in PBS; AR-INDIGO: 10 μM EthD-1 and 0.5 μM calcein AM in PBS). Cells were incubated for 2 h then imaged with Opera Phenix to capture green fluorescence for live cells (ex/em 494/517 nm) and red fluorescence for dead cells (ex/em 528/617 nm). Nuclear count was used to evaluate the number of dead cells per well.

### 3.10. Kinetic study of T in differentiated 3D HepaRG microtissues

To study the 3D HepaRG microtissue-mediated metabolism of T, HepaRG microtissues 2 days after seeding were exposed to 0, 30 or 100 nM T in DM (final concentration in 0.29 % DMSO) for 0 or 24 h (n = 5/group). The supernatant was collected at each of the time points into LoBind Eppendorf Tubes (Eppendorf, Hamburg, Germany) and stored at −80°C prior to LC-MS/MS analysis.

#### 3.10.1. Quantification of T and metabolites using LC-MS/MS

50 μL of sample and 150 μL of working internal standard (deuterated analogues of each analyte, specified below) were mixed and centrifuged (5 mins at 3000 rpm) prior to LC-MS analysis. The detection and quantification of T, AD, 6βOHT, and DHT in the supernatant following HepaRG microtissue incubation of T were accomplished using a Waters Xevo TQ-XS mass spectrometer, operating under positive electrospray ionization mode, connected to a Waters Acquity UPLC system. Chromatographic separation was performed using a Waters Acquity BEH C18 (50 ×2.1 mm, 1.7 μm particle size) column. The column and autosampler temperature were set at 40°C and 5°C, respectively. The injection volume was 5 μL at a flow rate of 0.5 mL/min. The mobile phase A consisted of water UPLC grade (Biosolve) + 0.1 % formic acid MilliQ water with 0.1 % and mobile phase B of ACN. The gradient was set at 10 % B for 0.3 min, followed by a linear increase to 100 % B from 0.3 – 1.5 min, 100 % B from 1.5 – 1.8 min, a linear decrease to 10 % B from 1.8 – 2.0 min, and re-equilibration from 2.0 to 2.5 min at 10 % B. The acquisition parameters of T, AD, 6βOHT, and DHT are summarized in Supplementary Materials S2. Calibration standards of T and AD were prepared as 0.25 – 50 nM in DM and for DHT and 6βOHT as 1.25 – 50 nM in DM. Working internal standards consisted of 10 nM ^13^C3-T, D_3_-DHT and D_3_-6βOHT in ACN, ^13^C3-T was also used as internal standard for AD. The recovery of the detection of the compounds was sufficient with minor adsorption (data not shown), indicating the adequacy of the LC-MS/MS method to detect the parent compound at t 0 h. For the quantification of the T and metabolite concentrations, a calibration graph of the analyte peak area / internal standard peak area was constructed and a 1/x2 weighting applied.

### 3.11. Two-chamber co-culture system with human liver and AR cells

To determine the androgenic response of T and DHT from the AR reporter assays in the absence or presence of hepatic biotransformation, AR-CALUX or AR-INDIGO cells were co-cultured with 3D HepaRG microtissues as described above. 5 H after seeding of the AR-INDIGO cells (D1 + 5 h), the cells were exposed to a concentration range of T and DHT. 24 H after seeding of the AR-CALUX cells (D2), the CALUX cells were exposed to a concentration range of T and DHT. Both cell lines were exposed for 24 h by replacing 100 μL of DM with DM that were spiked with T, DHT or vehicle control with the final DMSO concentration of 0.29 %. Our previous work showed that this incubation time was sufficient for solute diffusion in the system (Ip et al., 2024). To examine AR activation, the medium from both compartments was aspirated at each of the time points. The agarose hydrogels were removed using a pipette tip leaving only the AR-CALUX or AR-INDIGO cells at the bottom of the plate. The AR-INDIGO cells were lysed then luciferase detection was induced and measured. 100 μL of Luciferase Detection Reagent (LDR) consisting of Detection Buffer and Detection Substrate (1:1) was added to each well and plates were rested at room temperature for at least 5 min, avoiding shaking. When the cells were lysed, luminescence was measured using the Synergy H1 Hybrid Multimode (H1MM) Microplate Reader (BioTek® Instruments, Inc. Vermont, USA). The AR-CALUX cells were washed with 150 μL of 1:1 PBS:Milipore water solution then lysed with 30 μL lysis mix. The plates were placed for at least 30 min on a plate shaker (300 rpm) until cells were lysed after which luminescence was measured using the Synergy H1 Hybrid Multimode (H1MM) Microplate Reader wherein 100 μL Illuminate mix was automatically added to each well. The data are presented from three independent studies executed in technical triplicates.

### 3.12. Data analysis for kinetics experiments

JMP Pro software (JMP® Version 16. SAS Institute Inc., Cary, NC) was used for statistical analysis. The equal variance and normality of the data were tested with the Shapiro-Wilk test first prior to statistical analysis. The effect of growing the 3D HepaRG microtissues in DM without HC from D1 to D1 + 5 h and to D2 or in DM with HC from D1 to D3 was evaluated using Student’s two-sided *t*-test or its non-parametric equivalent with N = 4 and N = 6 for the 3D microtissues grown in DM without HC and DM with HC. The effect of the presence of 3D HepaRG microtissues on the AR reporter gene response at each exposed T and DHT concentration was evaluated using the Student’s two-sided *t*-test, Welch’s test or Median test, with N = 9 for each of the compound concentrations and N = 18 for the vehicle control. Statistical significance was set at P ≤ 0.05.

## 4. Results

### 4.1. Hydrocortisone interferes with the response of AR-INDIGO but not the AR-CALUX reporter cells

We observed significant interference of the luminescent signal from AR-INDIGO reporter cells when cultured in the complete HepaRG differentiation medium (DM) that consists of base medium supplemented with 10 % CDS-FBS, 5 μg/mL human insulin, 0.5 % DMSO, and hydrocortisone (HC). We identified HC as the agent that interferes with the AR-INDIGO reporter gene assay after conducting an elimination experiment by removing each of the medium additives one at a time (Supplementary Materials S3). HC does not interfere with the AR-CALUX reporter gene assay.

### 4.2. The characteristics of 3D HepaRG microtissues matured with or without hydrocortisone

HC is included in DM to support the growth and differentiation of the 3D HepaRG microtissues (Gripon et al., 2002). We investigated the effect of removing HC from the DM on 3D HepaRG microtissue formation and function with microscopy, gene expression and immunohistochemical analyses of relevant enzymes. Our previous work (Ip et al., 2024) reported an optimal maturation time for 3D HepaRG microtissues of 10 days for enzyme activation. However, to evaluate the optimal maturation time for compatibility with either AR-CALUX or AR-INDIGO reporter cells in the two-chamber co-culture system, a shorter 3D HepaRG maturation time of 3 days was examined.

**Fig. 3.**
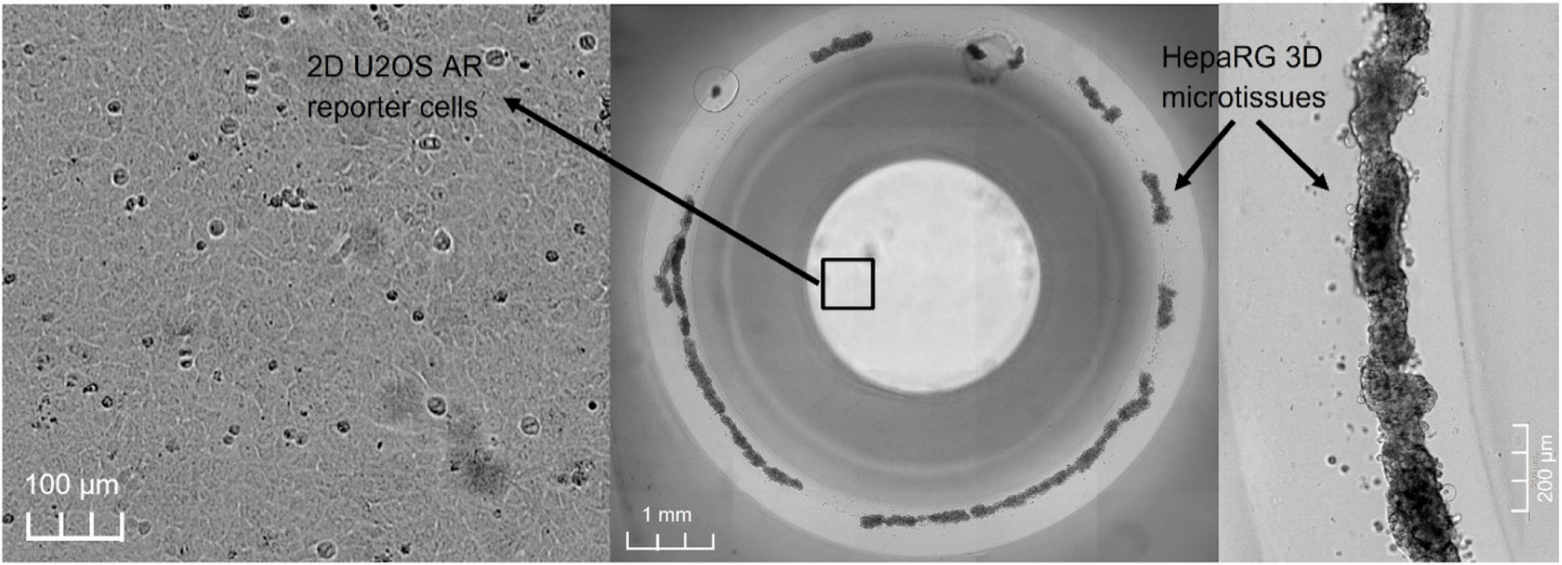
Live cell brightfield images at day 3 of 50,000 differentiated HepaRG cells forming 3D microtissues during culture in the outer ring-shaped trough and 8000 2D U2OS AR-CALUX cells in the central chamber of the two-chamber system.

Differentiated HepaRG cells were seeded in the outer ring-shaped trough on D0 and on D1 U2OS AR-CALUX cells were seeded in the central chamber. At D3, live cell brightfield images were obtained (Fig. 3), and both cell types were imaged at different planes using confocal microscopy. The 3D HepaRG microtissues were formed throughout the outer ring-shaped trough on day 3 similar to previous work (Ip et al., 2022) and the U2OS AR-CALUX cells formed a 2D monolayer in the central well on the bottom of the plate.

**Fig. 4.**
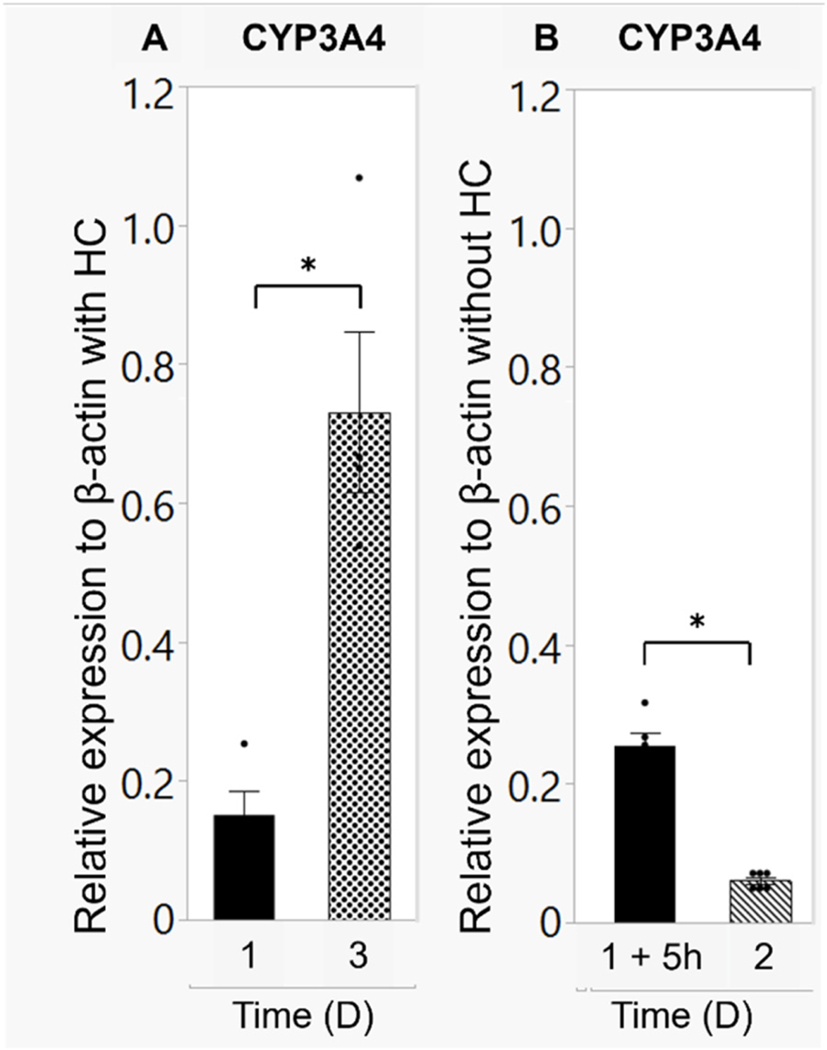
mRNA expression of CYP3A4 relative to β-actin in 3D HepaRG microtissues at D1 and D3 grown in DM supplemented with HC (Ip et al., 2024, A) and in 3D HepaRG microtissues switched to DM without HC at D1 and harvested at D1 plus 5 h (1 +5 h) and at D2 (B). The time points were significantly different (*P* ≤ 0.05) by Student’s t-test or its non-parametric equivalent.

We evaluated HepaRG 3D microtissue gene expression of CYP1A1, CYP1A2, CYP2B6, CYP2C9, CYP3A4, and UGT2B17at D1 + 5 h (for microtissues grown in MHTAP medium that contains HC then switched to DM without HC for 5 h) and D2 (DM without HC for 24 h) to examine the effects of removing HC. Removing HC at D1 significantly increased the gene expression of CYP1A1, CYP1A2, CYP2C9, and UGT2B17 (Supplementary Materials S4), and significantly decreased the expression of CYP3A4 (Fig. 4). The relative CYP enzyme mRNA expression levels in the 3D HepaRG microtissues grown in DM without HC for 2 days were CYP2C9 *>* CYP1A1 *>* CYP3A4, CYP1A2, CYP2B6, and UGT2B17. HepaRG microtissues grown in DM with HC from day 1 showed a significant increase in gene expression of all evaluated enzymes, including CYP3A4 (Fig. 4). The relative CYP enzyme mRNA expression levels in the 3D HepaRG microtissues grown in DM with HC for 3 days were CYP2C9 *>* UGT2B17 *>* CYP1A1, CYP2B6 *>* CYP3A4 *>* CYP1A2 (Ip et al., 2024). The mRNA expression levels of the respective enzymes in the 3D HepaRG microtissues grown in DM without HC for 2 days were lower compared to those in 3D HepaRG microtissues grown in DM with HC for 3 days (Fig. 4) (Ip et al., 2024).

### 4.3. Proportions of cells expressing CYP3A4 in 3D HepaRG microtissues with or without hydrocortisone are equivalent

**Fig. 5.**
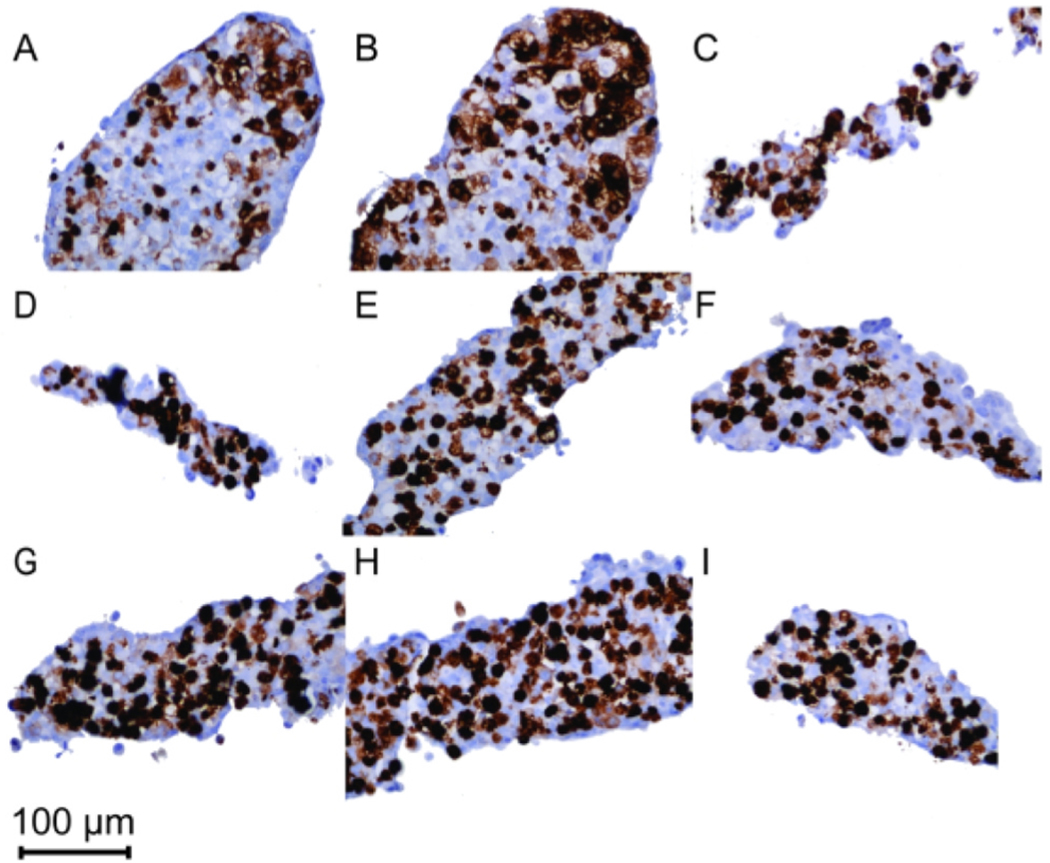
Immunohistochemical analysis of CYP3A4 in 3D HepaRG microtissues grown in DM supplemented with HC for 6 days (**A**) or 10 days (**B**) (Ip et al., 2024). Immunohistochemical analysis of CYP3A4 in 3D HepaRG microtissues grown in media without HC: For 1 day (**C**), exposed to vehicle control (0.29 % DMSO) for 24 h at D2 (**D**) or D3 (**E**), to 3 nM DHT for 24 h at D2 (**F**) or D3 (**G**), or 10 nM T for 24 h at D2 (**H**) or D3 (**I**) (n = 6/group).

Since the CYP mRNA transcript levels were low in the 3D HepaRG microtissues grown in DM without HC, we investigated the protein expression of CYP3A4 by immunohistochemical analysis (Fig. 5). These data were compared with CYP3A4 expression of 3D HepaRG microtissues grown for 6 or 10 days in DM supplemented with HC (Ip et al., 2024). Qualitatively, a similar proportion of cells within the 3D HepaRG microtissues expressed CYP3A4, independent of maturation time or HC exposure. Thus, despite having lower CYP3A4 mRNA transcript levels in 3D HepaRG microtissues grown in DM, not supplementing the DM with HC did not affect the proportion of cells expressing CYP3A4 from D1 to D3 (Fig. 5).

### 4.4. AR-CALUX and AR-INDIGO cells are viable when cultured in the two-chamber co-culture system

LIVE/DEAD staining showed that both reporter cell types were viable in the co-culture system over the course of 3 days without the presence of HC. Furthermore, the presence of 3D HepaRG microtissues did not affect their viability (Supplementary Materials S5).

### 4.5. 3D HepaRG microtissues metabolize testosterone

HepaRG 3D microtissues were incubated with T (0, 30 or 100 nM) for 24 h and the concentrations of T and its metabolites AD, 6βOHT, and DHT were measured using LC-MS/MS (Table 1). AD formation was detectable only in the presence of T and HepaRG 3D microtissues, and the amount of AD formed depended upon the starting concentration of T. This result complements prior work (Zhang et al., 2018) showing that AD was the major metabolite formed in *in vitro* when human liver cells were incubated with T. 6βOHT and DHT were not detected when HepaRG microtissues were exposed to these concentrations of T, likely because the amounts formed resulted in concentrations that fell below the limit of detection (LOD) of 1.25 nM. We compared the rates of T transformed and the formation rate of AD following incubation with HepaRG microtissues maturated for 3 days vs 10 days (Ip et al., 2024) and revealed that the metabolic rates are dependent on the T concentration added rather than the HepaRG maturation time (Table 2). At equivalent concentrations of T added, the AD formation rates are higher in HepaRG microtissues maturated for 3 days vs 10 days. This could indicate higher AD than T presence at the moment of luciferase transcription in the AR reporter cells during the co-culture assay, and thus a different *in vitro* AR-response.

### 4.6. 3D HepaRG microtissues reduce the androgenic responses of both AR gene reporter assays in the two-chamber co-culture system

**Fig. 6.**
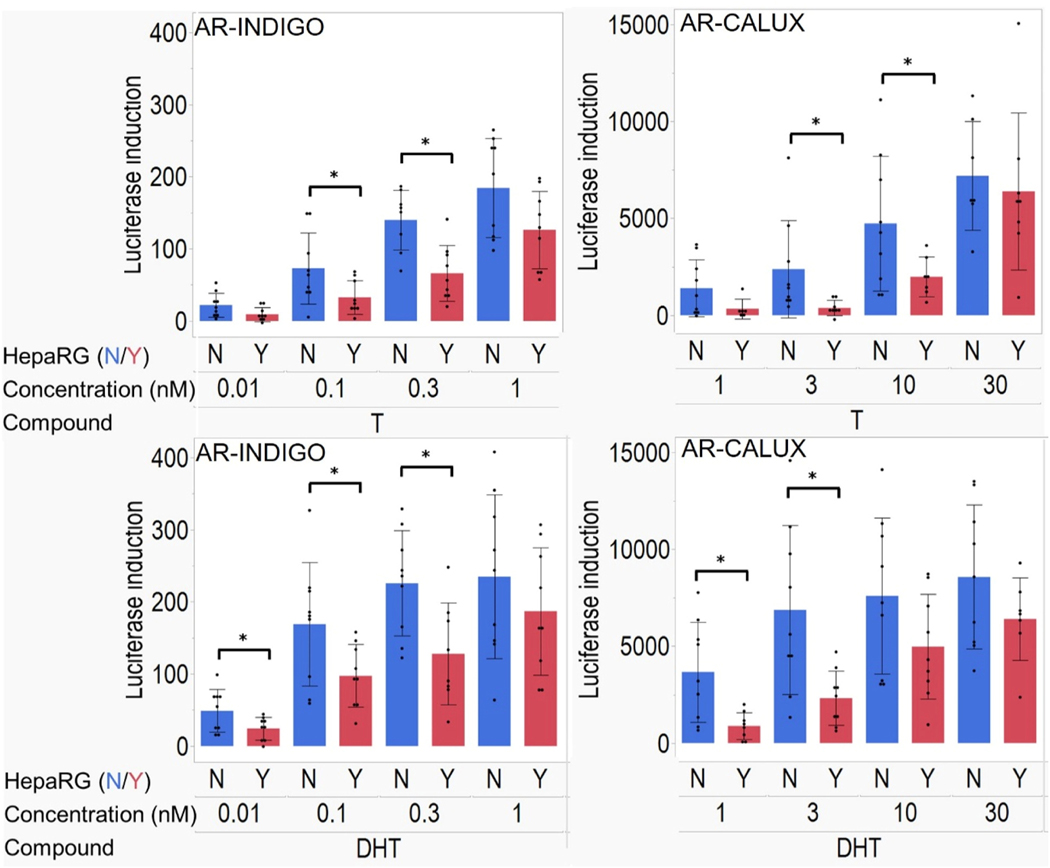
The concentration-dependent activity of T and DHT in the CV-1 AR-INDIGO and U2OS AR-CALUX reporter gene assay in the absence (blue bars (N)) and presence (red bars (Y)) of HepaRG microtissues using the two-chamber co-culture system with HepaRG and reporter cells. Data are depicted as mean ± SD and individual datapoints are depicted as filled circles in the graphs. Student’s *t*-test, Welch’s test or Median test, depending on equal variance and normality, were used to assess statistical significance between the responses observed in the absence and presence of HepaRG microtissues, *p* ≤ 0.05 (*).

The modified co-culture system, with a shorter HepaRG maturation time of 3 days and media without HC, was used to compare the androgenic activity of T and DHT in the AR-CALUX and AR-INDIGO reporter cells in the absence or presence of hepatic biotransformation. We observed different substrate concentration sensitivities between the AR-INDIGO and AR-CALUX assay in our two-chamber co-culture system with 3D HepaRG microtissues (Fig. 6). In the AR-INDIGO assay, we detected a significant difference in hepatic metabolism-mediated AR-response at 0.01–0.3 nM DHT and at 0.1 and 0.3 nM T. Whereas in the AR-CALUX assay, we saw a significant difference in hepatic metabolism-mediated AR-response at 1 and 3 nM DHT and 3 and 10 nM T. Both reporter gene assays were thus able to measure androgenic responses of T and DHT in the absence and presence of 3D HepaRG microtissues in the two-chamber co-culture system, showing the flexibility and reproducibility of the co-culture system.

Using the INDIGO reporter cells that are constitutively expressing the luciferase vector, we confirmed that increasing DHT or T concentrations in the presence of 3D HepaRG microtissue did not alter the AR response of the INDIGO reporter cells in the two-chamber the co-culture system (Supplementary Materials S6). Considering that the AR-INDIGO reporter cells detected an AR response at lower concentrations of T and DHT than the AR-CALUX cells, thus operating in a different dynamic range, we concluded that the AR-INDIGO cells were more sensitive in measuring androgenicity. However, the AR-INDIGO reporter cells generated lower luciferase signal intensity compared to the AR-CALUX reporter cells. The loss in AR response of AR-INDIGO reporter cells at higher doses of T an DHT is not due to loss of AR target tissues (Supplementary Materials S5) or loss in AR reporter functionality of the target tissues.

## 5. Discussion

In NGRA, in vitro bioactivity assays can be used to quantify toxicodynamic responses of chemicals deriving NAM PoDs which can be integrated in the safety assessment of human chemical exposure. However, under single cell culture conditions, processes such as hepatic biotransformation are rarely captured and, thus, the *in vitro*-derived dynamic response of the parent compound may not completely capture the pattern of activity in the human body (Coecke et al., 2006; Gu and Manautou, 2012; OECD DRP 97, 2008; Ooka et al., 2020). Ip et al. (2024) developed a human microphysiological co-culture system with 3D HepaRG microtissues and the AR-CALUX androgen gene reporter cells to assess androgenic responses of T in the presence of physiologically relevant human liver metabolism. In this study, the two-chamber co-culture system was further developed to test different AR reporter cell systems, evaluate different media conditions and HepaRG maturation times, as well as to examine DHT as an additional AR activating compound.

Many of the limitations of existing *in vitro* liver metabolism systems - high donor variability and less stable enzyme activities using PHHs or subcellular fractions like human liver S9 or minimal differentiation, such as with 2D HepaRG cells - can be overcome by using 3D HepaRG microtissues. 3D HepaRG microtissues have prolonged and robust liver characteristics, including both phase I and phase II metabolism enzyme expression, biliary excretion, and zonation characteristics as in the human liver (Gunness et al., 2013; Jackson et al., 2016; Leite et al., 2012; Ramaiahgari et al., 2017; Ip et al. 2024; Ooka et al., 2020), making them suitable as a physiologically relevant human *in vitro* liver model to measure the hepatic biotransformation of compounds. Another advantage of using 3D HepaRG microtissues to capture biotransformation in an *in vitro* bioactivity assay is that it utilizes human liver cells and chemical permeability through the cell membrane, thus reflecting metabolism in a more human-relevant manner as opposed to co-incubation with S9/microsomes (Mollergues et al., 2017; Sumida et al., 2001; van Vugt-Lussenburg et al., 2018). While 3D HepaRG microtissues may not fully replicate the complex physiology of the *in vivo* human liver, using the two-chamber co-culture system with metabolically competent HepaRG microtissues and target tissue provides more physiologically relevant insight into potential toxicity than *in vitro* systems using only target tissue without liver metabolism.

In the present study, we examined the flexibility and the robustness of the two-chamber co-culture assay (Ip et al., 2024) by testing another androgen gene reporter gene assay (AR-INDIGO) that required medium optimization by removing a key component in the HepaRG differentiation medium, as well as to evaluate the efficacy of the system with a shorter maturation time of the 3D HepaRG microtissue (from 10 to 3 days) to make the co-culture assay more compatible with different reporter cell systems. We also include DHT as another androgenic model compound to examine how hepatic biotransformation of AR substrates modulates AR activation.

The enzymes involved in the hepatic inactivation of T and DHT are mainly CYP3A4, HSD17B2, and UGT2B17, forming 6βOHT, AD, TG, and DHTG which are reduced in AR activity or inactive metabolites (Bhatt et al., 2018; Hashimoto et al., 2016; Li et al., 2019; Schiffer et al., 2018; Usmani and Tang, 2004; Zhang et al., 2018). The relative CYP enzyme mRNA expression levels in the 3D HepaRG microtissues grown in DM for 2 days were CYP2C9 *>* CYP1A1 *>* CYP3A4, CYP1A2, CYP2B6, and UGT2B17 which is different than the relative CYP enzyme mRNA expression levels in the *in vivo* human liver where the CYP3A4 level is the highest followed by CYP2C9 (Pelkonen et al., 2008). However, while gene expression of CYP3A4 in 3D HepaRG microtissues grown in DM at D2 was low, CYP3A4 immuno-staining revealed that CYP3A4 protein was present, and 3D HepaRG microtissues metabolized T to AD, indicating a difference in CYP3A4 mRNA and protein half-lives in our 3D HepaRG microtissues. The mRNA expression levels of metabolizing enzyme in the 3D HepaRG microtissues grown in DM without HC for 2 days were lower compared to those matured in DM with HC for 3 days (Ip et al., 2024), suggesting that HC has an important role in maintaining metabolizing enzyme gene expression.

In the present study, 6βOHT and DHT were below the detection limit following incubation of 30 and 100 nM T for 24 h in 3D HepaRG microtissues grown without HC, whereas treating 3D HepaRG microtissues matured for 10 days in DM containing HC with 200 μM T for 2 h resulted in formation of 0.035 nM 6βOHT/cell/h (Ip et al., 2024). We speculate that the difference between the two observations were due to the differences in i) the T concentration of 200 μM being 3 orders of magnitude higher than 100 nM, ii) the presence of HC in the DM, and iii) 3D HepaRG microtissue maturation for 10 days versus D3. Moreover, Phase II metabolism may be sufficiently rapid to deplete 6βOHT in the cells.

The observation that AD is the major metabolite formed *in vitro* may deviate from the *in vivo* situation due to the T concentration and incubation time. Transformation rates of T and formation rates of AD were similar following incubation with HepaRG microtissues maturated for 3 or 10 days and grown without and with HC, respectively, indicating that T-mediated metabolism is dependent on the T concentration added rather than HepaRG maturation time or HC presence. For a novel compound, more incubation time points and concentrations should be included to generate a more complete kinetic and metabolite profile of the parent compound over time, and to better relate the metabolite profile at 24 h used in the reporter gene assay to the metabolic pathway of the parent compound.

Metabolism of T and DHT by 3D HepaRG microtissues in our co-culture system led to reduced AR activation in two independent AR-reporter systems. This reduction, observed at low T concentrations, indicates effective metabolic conversion to less active metabolites, even below the LOD by high-quality mass spectrometry, and was not due to cell death.

The co-culture system was sufficiently flexible to identify the significantly different dynamic ranges of the target reporter assays. The differences in measuring significant alterations in androgen receptor response could be due to the differential sensitivity and dynamic ranges of the two AR-transfected cell lines (which have different luciferase reporter constructs), and the different experimental protocols used in the two systems (Sonneveld et al., 2005). Replicate variability in the observed AR responses could be addressed with robotics.

While complicated, the chemically induced *in vitro* AR response derived in the presence of 3D HepaRG microtissues is more informative and *in vivo-*relevant than the derived AR response in the absence of liver-mediated biotransformation. The system can assess the *in vitro* response of a compound in the presence of hepatic biotransformation to provide insight into whether bio(in)activation influences the corresponding bioactivity, and thus identifies potential metabolism modulation. The co-culture system detected concordant T- and DHT-mediated changes in the presence of hepatic biotransformation in both AR-CALUX and AR-INDIGO target assays. Thus, the co-culture system can integrate different reporter target cell systems and examine the bioactivity of different androgenic compounds and their metabolites. As an identification tool of potential metabolism modulation, one could determine whether metabolism increased, decreased, or left unchanged the target cell response, providing useful insight regarding a wide range of bioactivities. Integrating reporter data and metabolism systems into medium-throughput screening would help identify compounds that are (in)activated early on, enabling lower tier assessments without using animal experimentation in risk assessment.

Performing the two-chamber co-culture system for the desired identification of metabolism modulation thus has some requirements and limitations. First, preliminary optimization studies are needed to assess the target assay function under the two-chamber co-culture conditions, including cell viability over time, functionality in the presence of the 3D HepaRG microtissues, appropriate media conditions (Supplementary Materials S3), and the optimal reporter cell number. Finally, the two-chamber co-culture system using 2 cell types may not fully recapitulate the biotransformation mechanisms observed in the human body. For example, the HepaRG cell ratio to CALUX or INDIGO reporter gene cells in the two-chamber co-culture system is 6.25 and 5.55, respectively, which may not be indicative of the *in vivo* ratio between hepatic tissue mediating T and DHT inactivation and androgen responsive tissues. However, the system is designed to allow allometric scaling when the target organ type is defined, such as the kidney.

In conclusion, this two-chamber co-culture system with human liver and reporter cells is a flexible and robust tool that can rapidly determine *in vitro* toxicodynamic responses in the presence of hepatic biotransformation and contribute to the identification of potential metabolism modulation of compounds with (unknown) metabolites affecting the corresponding bioactivity, establishing more scientific confidence for the model.

## Supplementary Material

1

Appendix A. Supporting information

Supplementary data associated with this article can be found in the online version at doi:10.1016/j.toxlet.2025.08.014.

## Figures and Tables

**Table 1:** LC-MS/MS measured T and AD concentrations following incubation of 0, 30 or 100nMT with and without HepaRG microtissues for 0 and 24 h. Mean ± SD (n=5).

T added (nM)	w/o or with HepaRG microtissues	Incubation time (h)	T measured (nM)	AD measured (nM)
0	w/o HepaRG	24	<0.25	<0.25
0	HepaRG	24	<0.25	<0.25
30	w/o HepaRG	24	24.08 ± 0.76	<0.25
30	HepaRG	24	6.42 ± 0.37	11.88 ± 0.81
100	w/o HepaRG	24	72.60 ± 5.31	<0.25
100	HepaRG	24	24.37 ± 5.69	37.09 ± 4.03

**Table 2:** Rate of T transformed and the formation rate AD (pmol/hour/million cells) following incubation of 30 or 100 nM T for 24 h with HepaRG microtissues maturated for 3 days and incubation of 10, 30. 100 or1000 nM T for 24 h with HepaRG microtissues maturated for 10 days (Ip et al., 2024).

T added (nM)	Rate T transformed (pmol/hour/million cells) in 3 day-maturated HepaRG microtissues	Rate T transformed (pmol/hour/million cells) in 10 day-maturated HepaRG microtissues (Ip et al., 2024)	Formation rate AD (pmol/hour/million cells) in 3 day-maturated HepaRG microtissues	Formation rate AD (pmol/hour/million cells) in 10 day-maturated HepaRG microtissues (Ip et al., 2024)
10		1.29		0.42
30	4.19	4.56	2.82	1.30
100	11.45	13.18	8.81	3.94
1000		50.83		43.09

## Data Availability

Data will be made available on request.
