# Supplementary material for: Establishing scientific confidence in a two-chamber co-culture system to evaluate androgenic response in the presence of hepatic metabolism
Source: Toxicol Lett. Author manuscript; Available in PMC 2026 Jul 11. (PMC13354927; doi:10.1016/j.toxlet.2025.08.014)
Supplement: 1 [file NIHMS2182148-supplement-1.docx]

## Supplementary materials S1. Experimental procedures


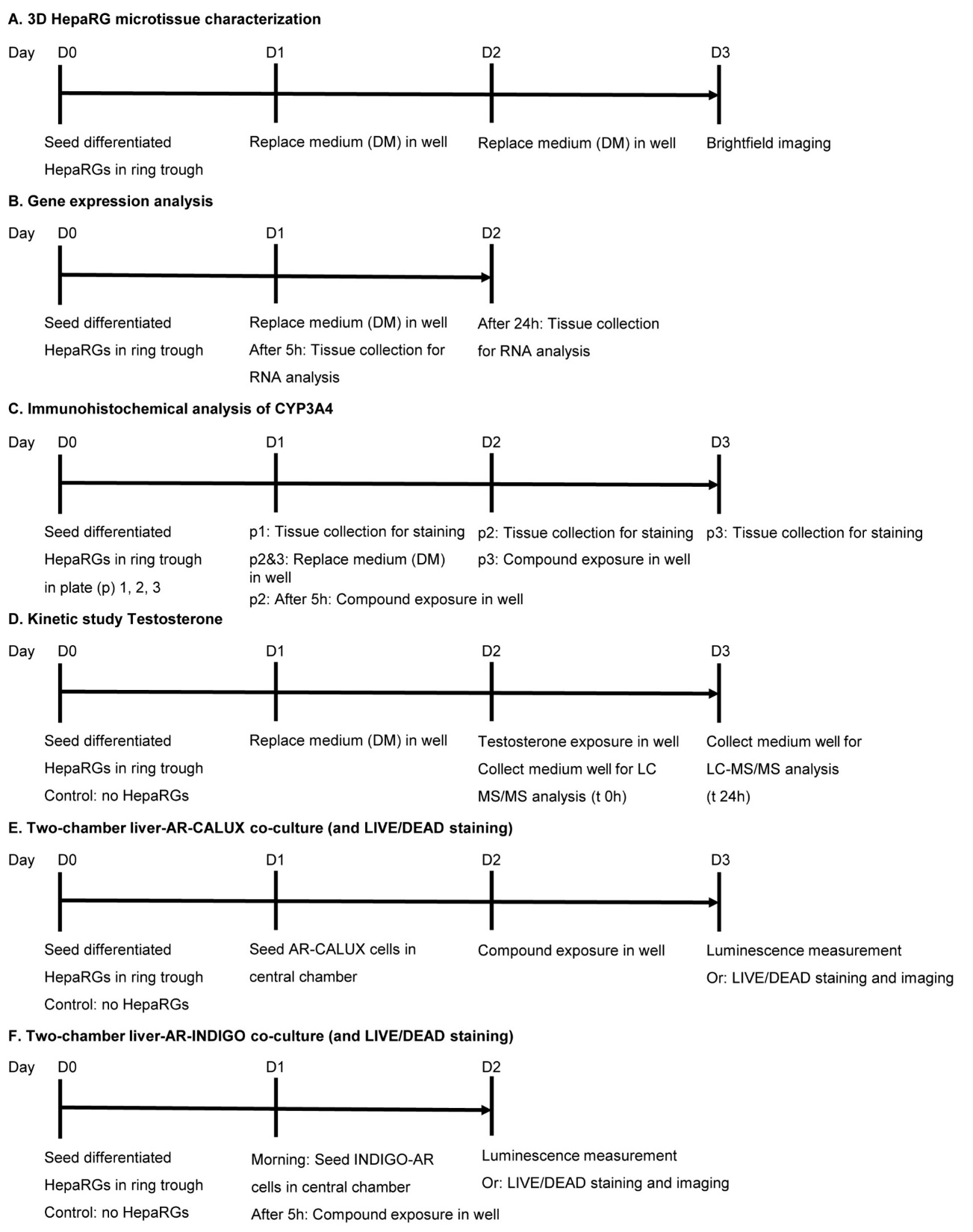


**Supplementary Figure S1.** Timelines of the experimental procedures. **A.** Characterizing the 3D HepaRG microtissues, **B.** Gene expression analysis, **C. I**mmunohistochemical analysis of CYP3A4, **D.** Performing the kinetic study incubating T with 3D HepaRG microtissues for 24 h, and performing the two-chamber co-culture system with human liver and AR-CALUX **(E)** or AR-INDIGO **(F)** cells to obtain concentration-dependent T- and DHT-mediated luciferase induction in the AR reporter gene assays in the absence or presence of hepatic metabolism, including performing the LIVE/DEAD staining of the U2OS AR-CALUX or CV-1 AR-INDIGO cells.

## Supplementary materials S2. LC-MS/MS acquisition parameters

| Compound | Precursor ion (m/z) | Product ion (m/z) | Collision energy (V) | Retention time (min) |
| --- | --- | --- | --- | --- |
| T | 289.1000 | 97.1000 | 22 | 1.87 |
| ^13^C3-T | 292.1597 | 100.1260 | 22 | 1.87 |
| DHT | 291.1600 | 255.2100 | 16 | 1.97 |
| DHT-d_3_ | 294.2000 | 258.2000 | 16 | 1.97 |
| 6βOHT | 305.1000 | 269.2100 | 14 | 1.58 |
| 6βOHT-d_3_ | 308.1000 | 272.2000 | 16 | 1.58 |
| AD | 287.10 | 97.0700 | 18 | 1.92 |

## Supplementary materials S3. Co-culture medium optimization

The 3D HepaRG microtissues were formed in MHTAP and differentiation medium (DM) consisting of base medium supplemented with 10% CDS-FBS, 5 μg/mL insulin, and 0.5% DMSO. In the co-culture system, the HepaRG and reporter gene cells resided in the same medium. Therefore, it was evaluated whether the components in the DM affected the reporter gene cells. Performing the standard AR-CALUX assay, the U2OS cells appeared to be fully functional when grown in DM (data not shown). However, the AR-INDIGO cells showed a lower signal and a high background response when the standard assay was grown using DM. Therefore, a standard AR-INDIGO assay was performed using 11-keto-DHT (11kDHT) as a model compound since this is the reference compound used in the standard INDIGO assay. 11kDHT has similar AR activity as DHT (Schiffer et al., 2018). The standard compound screening medium (CSM), MHTAP, full DM, and removing 1 component from the DM in each separate conditions, and the MHTAP medium to identify components affecting 11kDHT-mediated AR response was evaluated (Supplementary Fig. S3).


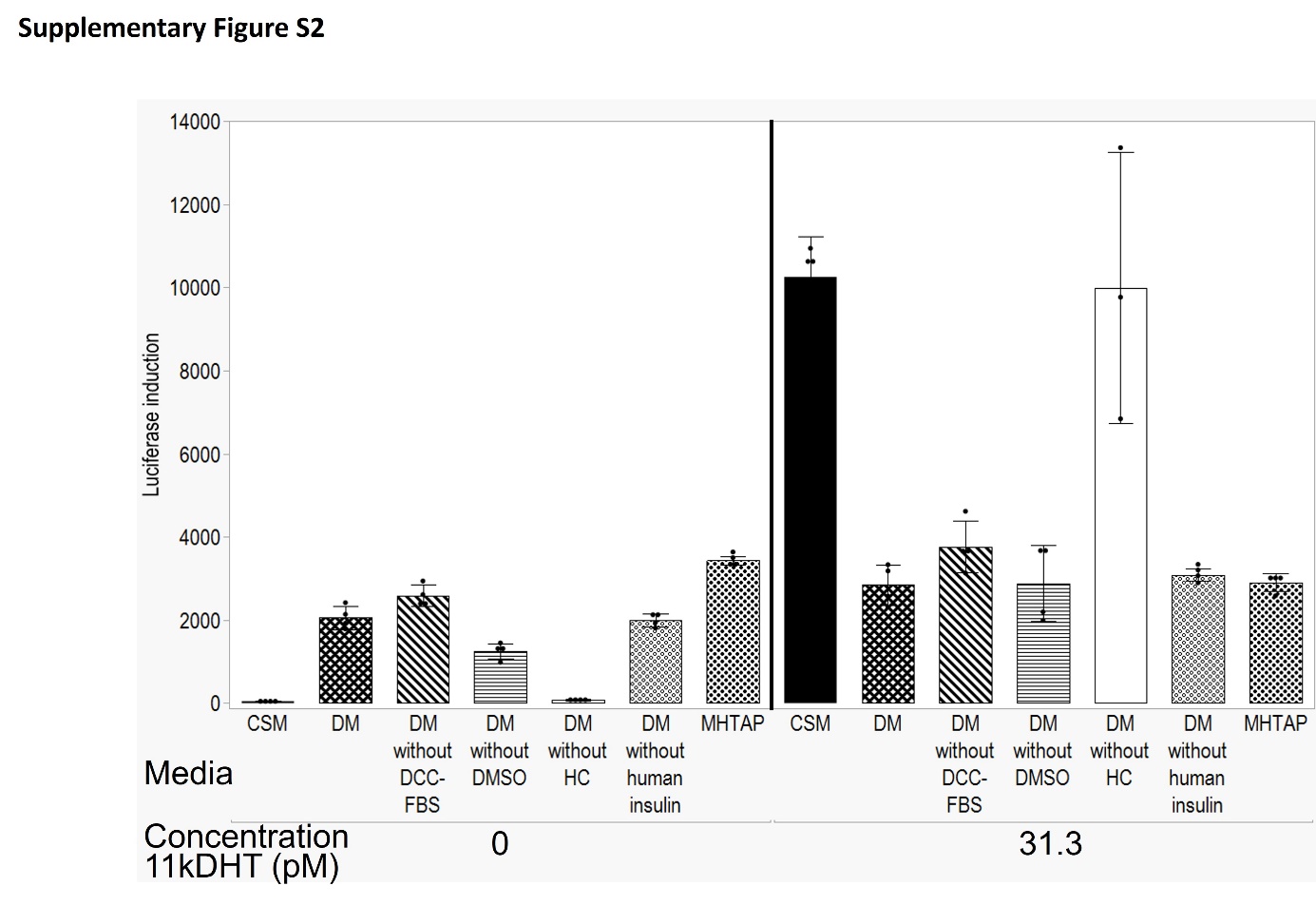


**Supplementary Figure S3.** 11kDHT-mediated AR response in the standard AR-INDIGO assay using different types of media. CSM = compound screening medium. DM = differentiation medium. Data are depicted as mean ± SD and individual datapoints are depicted as the dots in the graphs.

Comparing the background response at 0 nM 11kDHT using DM or DM without HC, removing HC from the DM decreased the background response. Removing HC from the DM increased the AR response at 31.3 pM 11kDHT. To synchronize methodologies using both reporter gene assays, HC was removed from the DM in the AR-CALUX and AR-INDIGO co-cultures.

## Supplementary Materials S4. mRNA expression of CYP1A1, CYP1A2, CYP2B6, CYP2C9, CYP3A4, and UGT2B17 in HepaRG microtissues


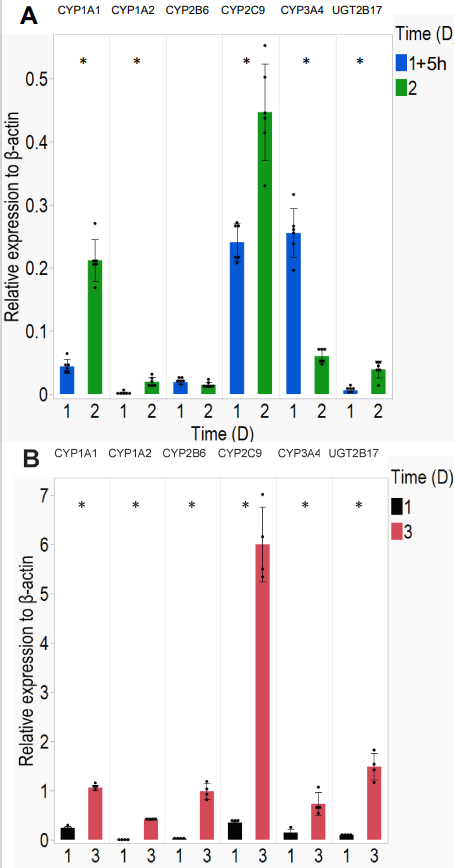


**Supplementary Figure S4.** mRNA expression of CYP1A1, CYP1A2, CYP2B6, CYP2C9, CYP3A4, and UGT2B17 relative to β-actin. A) Comparison of 3D HepaRG microtissues switched to DM without HC at D1 and harvested at D1 plus 5 hours (1+5h) and at D2. The expression levels for some CYPs significantly increased while others decreased or were unchanged between D1 plus 5 hours and D2. B) Comparison of 3D HepaRG microtissues at D1 and 3 grown in DM supplemented with HC (Ip et al. 2024). The expression of all the CYPs increased significantly between D1 and D3. *, p ≤ 0.05 by Student’s t-test or its non-parametric equivalent. P < 0.05.

## Supplementary Materials S5. LIVE/DEAD staining of U2OS and CV-1 cells in the central chamber of the two-chamber co-culture system

Supplementary Fig. S4.1 and S4.2 show the LIVE/DEAD staining of AR-CALUX cells and AR-INDIGO cells, respectively, in the central chamber in the two-chamber co-culture system with 3D HepaRG microtissues in the outer ring-shaped trough


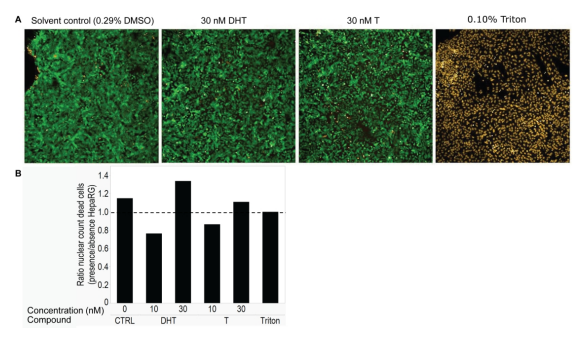


**Supplementary Figure S5.1** LIVE/DEAD staining of AR-CALUX cells in the central chamber at day 3 in the two-chamber co-culture system with 3D HepaRG microtissues in the outer ring-shaped trough. **A.** Calcein AM (green)- and EthD-1 (red)-stained U2OS cells exposed to solvent control (DMSO), 10 or 30 DHT or T or 0.10% triton. Live cells are stained by intracellular esterase activity converting calcein AM to the green fluorescent calcein. Dead cells are stained by EthD-1 that enters the cells with disrupted membranes and bind nucleic acids which intensifies its red fluorescence whereas the membrane of live protects from EthD-1 entrance. **B.** Ratio of the nuclear count of dead U2OS cells in the presence/absence of 3D HepaRG microtissues in the co-culture system exposed to 0, 10 or 30 nM T or DHT or 0.10% Triton.


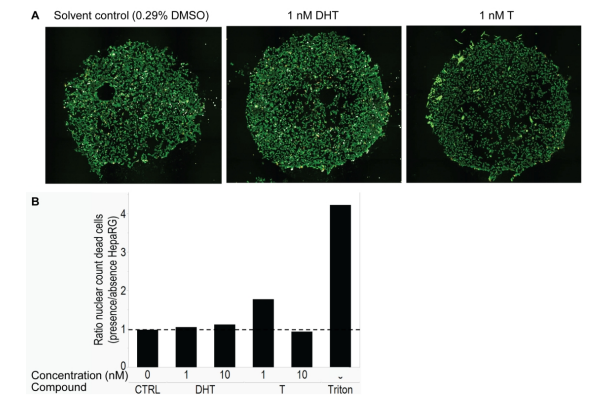


**Supplementary Figure S5.2** LIVE/DEAD staining of AR-INDIGO cells in the central chamber at day 2 in the two-chamber co-culture system with 3D HepaRG microtissues in the outer ring-shaped trough. **A.** Calcein AM (green)- and EthD-1 (red)-stained CV-1 cells exposed to solvent control (DMSO), 1 or 10 nM T or DHT or 0.10% triton. Live cells are stained by intracellular esterase activity converting calcein AM to the green fluorescent calcein. Dead cells are stained by EthD-1 that enters the cells with disrupted membranes and bind nucleic acids which intensifies its red fluorescence whereas the membrane of live protects from EthD-1 entrance. **B.** Ratio of the nuclear count of dead CV-1 cells in the presence/absence of 3D HepaRG microtissues in the co-culture system exposed to 0, 1 or 10 nM T or DHT or 0.10% Triton

## Supplementary Materials S6. Two-chamber liver-Constitutively LUC-INDIGO co-culture

Supplementary Fig. S6 shows the concentration-dependent agonistic activity of 11kDHT in INDIGO cells constitutively expressing luciferase.


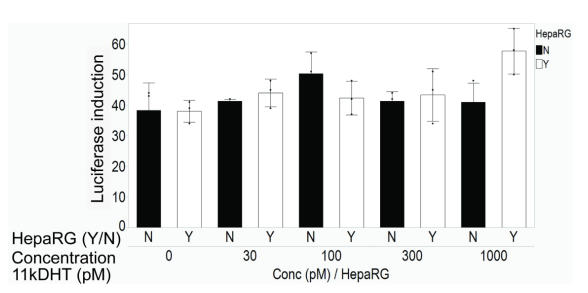


**Supplementary Figure S6.** The concentration-dependent agonistic activity of 11kDHT (0-1000 pM) in INDIGO cells constitutively expressing luciferase in the absence (black bars (N)) and presence (white bars (Y)) of 3D HepaRG microtissues. Data are depicted as mean ± SD.

## References

Schiffer, L., Arlt, W., & Storbeck, K. H. (2018). Intracrine androgen biosynthesis, metabolism and action revisited. In *Molecular and Cellular Endocrinology* (Vol. 465). https://doi.org/10.1016/j.mce.2017.08.016
